# Insects for breakfast and whales for dinner: the diet and body condition of dingoes on Fraser Island (K’gari)

**DOI:** 10.1038/srep23469

**Published:** 2016-03-24

**Authors:** Linda Behrendorff, Luke K.-P. Leung, Allan McKinnon, Jon Hanger, Grant Belonje, Jenna Tapply, Darryl Jones, Benjamin L. Allen

**Affiliations:** 1 The University of Queensland, School of Agriculture and Food Sciences, Gatton, Queensland 4343, Australia; 2 Queensland Parks and Wildlife Service, Department of National Parks, Sport and Racing, Fraser Island, Queensland 4581, Australia; 3 Department of Environmental Heritage Protection, Threatened Species Unit, Moggill, Queensland 4070, Australia; 4 Endeavour Veterinary Ecology, Toorbul, Queensland 4510, Australia; 5 Fraser Coast Veterinary Services, Maryborough, Queensland 4650, Australia; 6 Griffith University, Environmental Futures Research Institute, Nathan, Queensland 4111, Australia; 7 The University of Southern Queensland, Institute for Agriculture and the Environment, Toowoomba, Queensland 4350, Australia

## Abstract

Top-predators play stabilising roles in island food webs, including Fraser Island, Australia. Subsidising generalist predators with human-sourced food could disrupt this balance, but has been proposed to improve the overall health of the island’s dingo (*Canis lupus dingo*) population, which is allegedly ‘starving’ or in ‘poor condition’. We assess this hypothesis by describing the diet and health of dingoes on Fraser Island from datasets collected between 2001 and 2015. Medium-sized mammals (such as bandicoots) and fish were the most common food items detected in dingo scat records. Stomach contents records revealed additional information on diet, such as the occurrence of human-sourced foods. Trail camera records highlighted dingo utilisation of stranded marine fauna, particularly turtles and whales. Mean adult body weights were higher than the national average, body condition scores and abundant-excessive fat reserves indicated a generally ideal-heavy physical condition, and parasite loads were low and comparable to other dingo populations. These data do not support hypotheses that Fraser Island dingoes have restricted diets or are in poor physical condition. Rather, they indicate that dingoes on Fraser Island are capable of exploiting a diverse array of food sources which contributes to the vast majority of dingoes being of good-excellent physical condition.

Studies investigating the diet and foraging behaviour of top-predators are fundamental to our understanding of predator-prey relationships and the forces that shape terrestrial ecosystems. Large predators (such as ursids or felids) typically have high energy requirements and are often required to consume a relatively narrow range of large-bodied prey to survive^1^,^2^,^3^. Smaller predators typically have more flexible and broader dietary requirements. Mid-sized predators or mesopredators likewise exhibit flexible foraging behaviours that can reflect those of either large or small predators^4^. Diet also affects predator health and nutrition, and the reproductive capacity of individuals and populations. Knowledge of predator diets can therefore provide valuable insights into the impacts of predators on prey and ecosystems^5^,^6^, the detection of rare or threatened fauna^7^,^8^,^9^, and the general health, condition and energy requirements of predator populations^3^,^10^. This information may be particularly important for small, island populations of conservation concern.

The dingo (*Canis lupus dingo* and hybrids; alternative suggested nomenclature is found elsewhere^11^,^12^) is the largest terrestrial predator on mainland Australia and many offshore islands^13^,^14^ but are classical mesopredators with broad, generalist diets and flexible social and feeding ecology^15^. Dingoes have a long evolutionary association with humans^16^,^17^,^18^,^19^,^20^,^21^ and readily exploit anthropogenic food subsidies, sometimes becoming commensal with humans^22^,^23^,^24^. Their main prey are small-medium sized mammals, although larger prey (including *Macropus* spp. and livestock) are important sources of food in some areas^14^,^25^. Dingoes can suppress populations of their prey^5^,^26^,^27^,^28^, and free-ranging individuals require ~1 kg of food/day for an average-sized dingo to maintain condition^29^. Prey availability is, therefore, likely to be one of the most important drivers of dingo abundance and ecological function^24^,^26^,^30^,^31^. Island populations of dingoes subsidized by humans may be particularly sensitive to changes in prey or food availability, which may influence their overall body condition and other aspects of fitness, as is known for small island populations of the closely-related grey wolf (*Canis lupus*)^32^,^33^.

Dingoes on Fraser Island (K’gari), which are known as wangari by the aboriginal Butchulla people^34^, are an important element of the island’s cultural, ecological and conservation values^35^,^36^. There are approximately 100–200 dingoes on the island at any one time^37^,^38^, and despite great public interest, their ecology is poorly understood^35^,^39^,^40^,^41^. For example, there are over 34,000 records of dingo diets from across Australia^5^; however, no study of the diet of dingoes on Fraser Island has ever been published, although some limited unpublished reports are available^42^,^43^ (Table 1).

One of the most common topics of public interest and contention is associated with the diet and body condition of dingoes on Fraser Island. Media reports and other anecdotal information frequently describe dingoes as starving, endangered and/or declining due to lack of food resources, and of generally poor body condition. In public discourse these factors are often attributed to alleged mismanagement of dingoes by government agencies^44^,^45^,^46^,^47^,^35^ that restrict dingo access to rubbish dumps and major townships and prohibit dingo-feeding by humans. These actions have been undertaken as part of ongoing efforts to conserve the dingoes’ natural wild behaviour and reduce the safety risk to humans from dingo attacks^35^,^36^,^48^. A lack of available data on Fraser Island dingo ecology fosters these contentions.

Assessing the hypotheses that dingoes on Fraser Island have restricted diets, or are starving and are of poor physical condition merely requires a general description of what dingoes eat, and a description of dingoes’ health status, such as body weight, body condition score, fat reserves, parasite loads etc. Therefore, we used empirical data obtained from a variety of different data sources (including dingo scats, stomach contents and trail camera images) to investigate dingo diet and body condition. We also present data on dingo weights, body condition scores, internal fat reserves and parasite prevalence obtained from necropsy reports and other records collected during routine dingo trapping and ear-tagging programs to compliment these datasets. Our aims were to describe the prey and food items consumed by dingoes, and the resulting body condition and physical health of dingoes on the island to address speculation over dingoes’ diet and health status.

## Results

### Dingo diet

**Figure 1:**
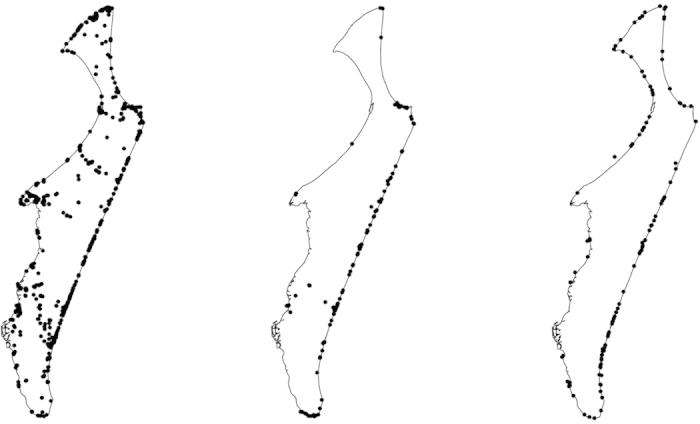
The location of (left) scat samples, (middle) stomach samples and (right) marine strandings (January 2011 to December 2014) described in this study. Maps created new by the authors in ArcGIS v9.3 and 10.1 (ESRI Inc.).

**Figure 2:**
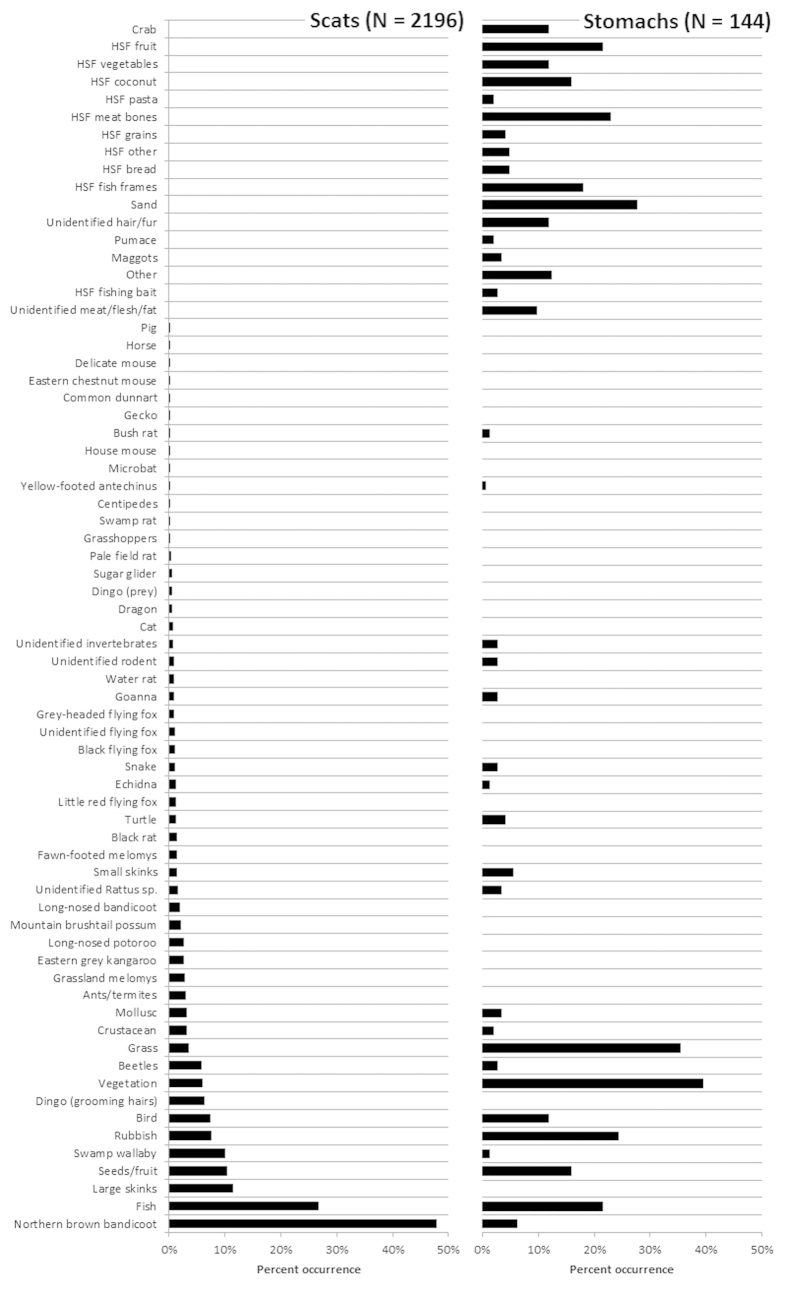
Percent occurrence of food items found in (left) 2,196 dingo scats collected mostly between March 2011 and October 2014, and (right) 144 dingo stomachs and intestines collected between August 2001 and January 2015 (HSF = Human-sourced food).

We collected 2,196 dingo scats, 144 stomach contents, and from >30,000 camera records we identified ~50 separate photographic events of dingoes carrying some food item or feeding on stranded marine mammals and reptiles from across the entire island (Fig. 1). Between 3 and 365 scats (mean = 84) were collected in each section of the island, with eight sections not yielding at least 30 scats. Mammals were the most frequently occurring food group in scats, followed by fish and reptiles (Table 1 and Fig. 2). The most frequently occurring food items in scats were northern brown bandicoots (*Isoodon macrourus*; 47.9%), followed by fish (26.8%) and large skinks (e.g. *Tiliqua scincoides*, *Egernia major*, *Hemisphaeriodon gerrardii*; 11.5%). Besides swamp wallabies (which occurred in 10.1% of scats; see also Fig. S1 or www.frasercoastchronicle.com.au/news/fraser-dingo-attacking-wallaby-surf-caught-video/1635907), other macropods were infrequently detected (e.g. eastern grey kangaroos *Macropus giganteous*, 2.6%; long-nosed potoroo *Potorous tridactylus*, 2.6%). The remains of large mammals occurred at very low frequencies (e.g. feral pigs *Sus scrofa*, <0.1%; feral horses *Equus callabus*, <0.1%). Human-sourced non-digestible items (e.g. plastic food wrappers, tin foil) occurred in 7.5% of scats (Fig. 2).

Stomach samples likewise indicated the importance of fish (21.5% occurrence; Fig. 2). However, the most frequently occurring food items in stomachs were vegetation (39.6%), grass (35.4%) and rubbish (24.3%). Much of the fruit (21.5%) detected were assumed to be *Dianella* spp, *Austromyrtus dulcis* and/or *Scaevola calendulcea*. A variety of other human-sourced food items were also found in stomachs, but not found in scats (Fig. 2 and Table S1). These included vegetables (11.8%), pasta (2.1%), bread (4.9%) and meat bones (22.9%).

**Figure 3:**
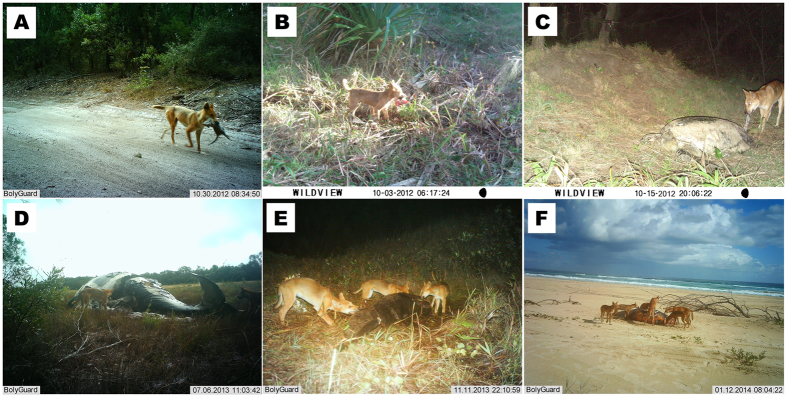
Trail camera images of dingoes (**A**) carrying a potoroo (*Potorous tridactylus*), (**B**) carrying human-sourced food, (**C**) feeding on a dead green turtle (*Chelonia mydas*), (**D**) feeding on an orca (*Orcinus orca*), (**E**) feeding on a dolphin (*Tursoips* spp.) and (**F**) feeding on a dugong (*Dugong dugon*).

Photographic images confirmed that potoroos, bandicoots, possums (*Trichosurus* sp.), short-beaked echidnas (*Tachyglossus aculeatus*) and other fauna are eaten by dingoes (Fig. 3 and Table S1). Dingoes were also observed carrying rubbish (Fig. 3). Cameras opportunistically placed to monitor dingo activity at stranded marine mammals and reptiles also recorded dingoes frequently feeding on a variety of whales, dolphins and turtles (Table 2), which regularly wash ashore (Fig. S1). Groups of dingoes resident in areas where marine items washed ashore, and also individual dingoes from other parts of the island (identified by unique ear-tags or individual sock and tail markings or scars observable on-camera) repeatedly returned to feed on larger items, such as dugongs, for up to nine weeks (and perhaps longer) before cameras were removed (Fig. 3).

The following incidental items were also observed first-hand to be eaten (whole or in part) by dingoes: human feaces, underwear, hats, a variety of different shoes, fish hooks, iPods, alcohol bladders, beer and soft drink cans, steel wool, plastic containers and onions.

### Dingo body weight and condition

**Figure 4:**
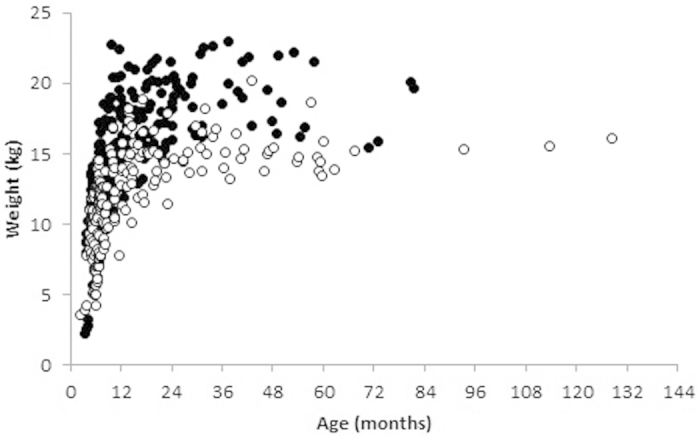
Body weights of male (solid marks, N = 251) and female (hollow marks, N = 204) dingoes captured, euthanized or found dead on Fraser Island between August 2001 and January 2015.

Mean adult body weight for dingoes >12 months old was 14.8 kg for females (N = 81) and 18.0 kg for males (N = 101), or 16.6 kg overall (N = 182). Overall body weight of all dingoes, inclusive of all ages and sexes was 13.6 kg (N = 455). Plotting body weight against age indicated that dingoes reach mean adult weight by approximatley 12 months of age (Fig. 4). On only one occasion did a dingo >12 months old weigh less than 10 kg.

**Figure 5:**
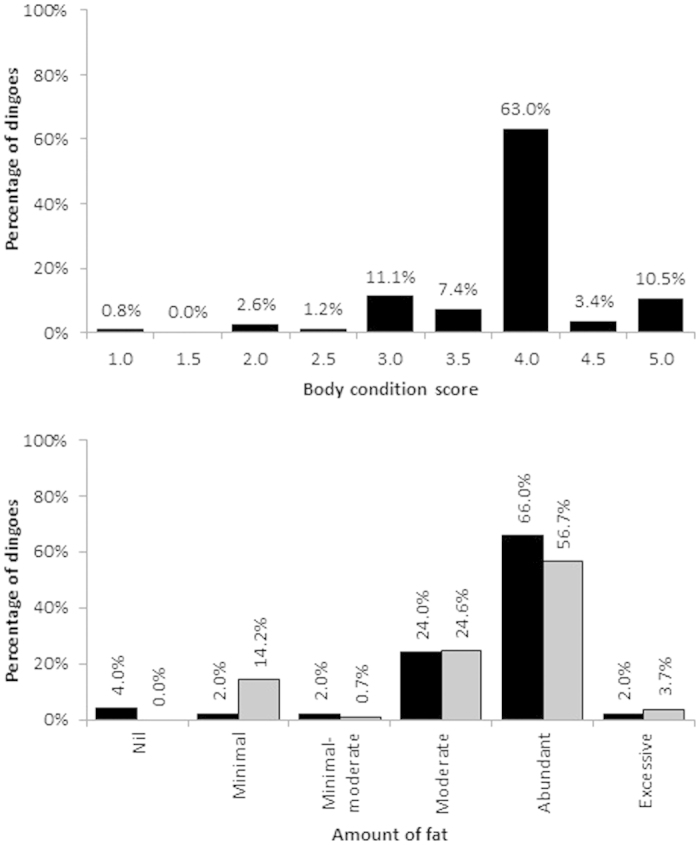
Body condition score (top, N = 503) and fat reserves (bottom; black bars = peri-renal, N = 50; grey bars = mesentery, N = 134) of dingoes either captured, euthanized or found dead on Fraser Island between July 2003 and January 2015.

**Figure 6:**
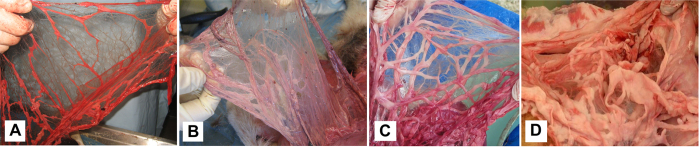
Veterinary-assessed examples of (**A**) minimal, (**B**) moderate, (**C**) abundant and (**D**) excessive mesentery fat reserves of dingoes on Fraser Island.

The mean body score of dingoes was 3.9 (SE 0.03, range 1–5, N = 503; Fig. 5), indicative of ‘ideal’ to ‘heavy’ physical condition. Over 10% of dingoes sampled had a body score of 5 (or ‘grossly obese’), and 62.9% of dingoes had a body score of 4 (‘heavy’), whereas a total of 5.6% of dingoes had a body score of 2.5 (‘underweight’) or less. Fat stores around mesentery (N = 134) and peri-renal areas (N = 50) of necropsied dingoes indicated that over 60% of dingoes had abundant fat in these areas; over 80% of dingoes had moderate-abundant fat stores or higher (Figs 5 and 6). Mean thickness of abdominal subcutaneous fat was 2.2 mm (SE 0.1 mm, range 0–8 mm, N = 133). On average, 30.5% (SE 1.9%, range = 0–100% coverage, N = 128) of hearts were covered with 3.0 mm (SE 0.2 mm, range 0–10 mm, N = 128) of fat, and 34.0% (SE 2.1%, range 0–100% coverage, N = 115) of kidneys were covered with 3.9 mm (SE 0.3 mm, range 0–20 mm, N = 115) of fat.

### Ecto- and endoparasites

Almost 95% (N = 773) of dingoes examined were free from ectoparasites (Table 3). The prevalence of fleas, ticks (including *Ixodes holocyclus* and *Rhipicephalus (Boophilus) microplus*) and mange (*Sarcoptes scabiei*) was <4.0%. Endoparasites detected during necropsy included heartworm (6.7%, N = 149) and flukes (5.4%, N = 111). Endoparasites detected in analysed faecal samples included *Ancylostoma* spp. (15.9%, N = 44) and *Toxocara canis* (2.3%, N = 44); sporocysts of *Monocystis* spp. were also recorded, indicating that dingoes also eat earthworms infected with Monocystis. Hookworms (in total) were found in 88.6% (N = 44) of fresh faeces. A separate study assessing intesinal parasites from 18 dingoes collected between December 2003 and April 2005 also recovered two specimens of trematode or fluke (*Haplorchinae* spp., probably *H. sensu*), usually found in fish and seabirds, from one dingo^49^.

## Discussion

This study confirmed that dingoes on Fraser Island consume a wide variety of food items, ranging in size from insects to whales (Table 2 and Fig. 2). However, as demonstrated in previous studies undertaken in eastern Australia^7^,^14^,^31^,^50^,^51^,^52^,^53^,^54^, medium-sized mammals (such as bandicoots) were the most common item detected in scats. The relatively high occurrence of fish (26.8%) has not been commonly reported for dingoes^13^, but the relatively high occurrence of large skinks and swamp wallabies (*Wallabia bicolour*) is common in other mainland locations^23^,^52^,^55^. Stomach contents revealed additional information that was not captured in scat data, such as the occurrence of human-sourced foods including pasta, bread, meat bones, chicken and fruit (Fig. 2 and Table S1). Likewise, camera data further highlighted dingo utilisation of stranded marine fauna, particularly turtles and whales (Table 2 and Fig. 3). Given the utilisation of these food items in Fraser Island dingo diets, their mean adult body weights (16.6 kg; Fig. 4) are higher than the national average (15.7 kg^4^), body condition scores and abundant-excessive fat reserves are indicative of generally ideal-heavy physical condition (Fig. 5; Fig. S1), and parasite loads are low and comparable to other dingo populations^22^,^56^,^57^ (Table 3). These data do not support hypotheses that Fraser Island dingoes are starving, have restricted diets or are in poor physical condition. Rather, they indicate that dingoes on Fraser Island are capable of exploiting a diverse array of food sources which contributes to the vast majority of dingoes being of good-excellent physical condition.

Given that our island-wide sampling protocols allowed for one of the largest collections of dingo scats ever recorded for a given site^5^, we are confident that our results are representative of the entire dingo population on the island. Results from previous unpublished, Fraser Island studies broadly reflect our findings (Table 1), although we cannot reliably compare most of them due to sample size differences, their spatially restricted sampling, and differences in analytical methodology. Rare and threatened fauna (such as the long-nosed potoroo *Potorous tridactylus* and eastern chestnut mouse *Pseudomys gracillicaudatus*) also featured in scat and or camera records (Table S1), confirming that these prey species are still present on the island. However, given prey of this size are preferred by dingoes^5^,^13^, the relative infrequency that they were recorded may be indicative of dingo suppression of these threatened prey species or just preferences for other available food^58^, amongst other possibilities. The same might also apply to kangaroos. The presence of feral pig, feral cat and feral horse remains, which were also detected incidentally in dingo scats (Fig. 2), likewise confirm their presence on the island. Pigs are a common occurrence in dingo diets in northern Australia^25^,^50^, although cats and horses are not common food items for dingoes anywhere^13^,^59^.

One of the more noteworthy differences between our results and those of previous studies is the apparent substantial decline of human-sourced food items in dingo diets over the last 20 years (Table 1). The study of Twyford^60^ occurred when several open rubbish dumps were available and exploited by dingoes^61^,^62^. Major townships and campgrounds on the island (places where human feeding of dingoes was common) were also unfenced at that time. Since then, all open rubbish dumps have been closed to dingoes, which are also excluded from several major townships and campgrounds^35^. Public education and compliance activities have also increased during this period, with the goal of reducing negative dingo-human interactions and preventing dingoes from gaining access to food subsidies, which are well-known to disrupt natural ecological processes and produce negative effects on biodiversity^23^,^24^,^63^,^64^. Our data are consistent with the view that these non-lethal management actions have reduced the availability of human-sourced food items to dingoes, and that they are now relying on more natural food sources. Such a return to more natural foraging behaviours might in turn reduce the risk of negative human-dingo interactions and the need for specific management actions necessary to address issues of human safety. However, the relatively high occurrence of fish (Fig. 2) could be indicative of continued subsidising^43^, although scat data alone are known to provide only limited information on true dingo diet^58^.

The additional stomach contents we analysed were particularly valuable for elucidating the occurrence of human-sourced foods not detected in scat samples (Fig. 2 and Table S1). Many of the items detected in stomachs (such as bread, pasta, fruit and chicken flesh) would likely be fully digested and not detectable in scats using the analytical procedures employed^65^,^66^. Such processed foods are typically high in energy and may be an important, albeit relatively infrequent source of nutrition for dingoes. However, given that our stomach samples originate primarily from euthanized or habituated dingoes in or around high-use visitor areas along the eastern beach^38^ (Fig. 1), it is possible that the reported high occurrence of such human-sourced foods in stomachs may be an artefact of our sampling and may not represent the wider dingo population on the island. In any event, such completely unnatural food sources should not be available to dingoes if the management goal is to ‘keep them wild’ and reduce the possibility of negative dingo-human interactions^67^.

Additional data from remote cameras revealed further important dingo food sources not recorded or identified in either scat or stomach analyses (Table S1). Like crocodile eggs^68^,^69^, turtles and particularly their eggs are well-known to be exploited by dingoes and other wild dogs^70^,^71^,^72^,^73^. Dingoes have been responsible for 50–86% of annual marine turtle nest failures on Fraser Island^74^, and also attack nesting adult female sea turtles (L. Behrendorff, unpublished data). Stranded whales and other marine mammals are infrequently reported in dingo diets^13^. The value of marine food resources cannot be underestimated given that large marine mammals provide several months of available food to resident and non-resident dingoes and such strandings are relatively frequent (Fig. S2). Photographs taken at one stranded whale between June and August 2010 show multiple dingoes from resident and nearby packs frequently returning to feed on the decaying carcass (Fig. 3), along with an individual from a distant pack up to 20 km away. Bivalves (or ‘pippies’), and stranded or washed-ashore sea snakes, sharks, rays, fish, and regular migratory bird stranding events^75^ (including short-tailed shearwaters *Puffinus tenuirostris* and Australasian gannets *Morus serrator*) also contribute to available food sources for dingoes and other scavengers on Fraser Island, although these strandings are not formally recorded as they are for marine turtles and mammals (available at www.ehp.qld.gov.au/wildlife/caring-for-wildlife/strandnet-reports.html). Most recorded strandings occur on the eastern beach where they are easily observed (Fig. 1), but due to the remoteness of the western beach areas, which are rarely frequented by people, the reported strandings likely underestimate the true number of stranded marine fauna available to dingoes. At least 75% of dingo packs on the island have direct beach access^38^, suggesting that marine strandings may be a particularly important, yet often unrecognised source of food for dingoes and other beach scavengers^76^. More work is required to evaluate dingo reliance on this important food resource.

That Fraser Island dingoes are heavier than mainland dingoes and frequently have abundant-excessive amounts of internal fat reserves (Figs 5 and 6) suggests that the food items dingoes consume are sufficient to produce and maintain a dingo population in good physical condition. To our knowledge, our sample sizes of body weights (N = 455), body condition scores (N = 503) and fat reserves (N = 134) are the largest of any dingo population in Australia. Thus, these data most likely represent the best available data on dingo weight and body condition for any given site. Underweight or skinny dingoes are sometimes observed on the island^45^, but most of these likely represent either the ‘doomed surplus’^77^ of excess or socially excluded individuals, females that have recently lactated and raised litters, sick or diseased animals, or those suffering temporary nutritional stress during normal periods of food shortage^78^ (Fig. S1). Dingoes with very high body scores were observed more frequently than those with low scores (Fig. 5). The relationship between mass or body condition and fitness is not linear^79^,^80^, or in other words, heavier or fatter dingoes might not be healthier or fitter dingoes. The mean body condition score of 3.9 (out of 5) is in excess of what is considered ideal for domestic dogs^81^,^82^, suggesting that Fraser Island dingoes are either overweight or that ideal or optimal scores for dingoes may be slightly different than domestic dogs. Moreover, dingoes and other predators have excess digestive capacity, do not eat each day, and regularly endure periods of several days without food or water^10^,^83^, suggesting that variable body condition scores and fat reserves might be expected in and between individuals under normal conditions. These normal and natural phenomena are associated with most wild populations of dingoes and other wildlife^80^, and are consistent with the view that Fraser Island dingoes, in general, are of average to above-average body weight and good-excellent physical condition.

Parasite levels were low and similar to mainland populations^13^,^56^ (Table 3), with the exception that hydatids (*Echinococcus granulosus*) are notably absent in Fraser Island dingoes^49^. However, the presence of heartworm (*Dirofilaria immitis*) may be of concern for the consevation of dingo populations if these worms become more prevalent. Heartworm is known to have previously decimated wild dingo populations in the Northern Territory^13^. Heartworm is difficult to detect during necropsy in young animals and can only be detected in blood samples after approximately six months post infection^84^. Adult dingoes >2 years of age are infrequently available for necropsy^38^, which means our results may underestimate the true prevalence of heartworm on the island. Future testing of blood samples collected during routine trapping and ear-tagging procedures on the island would greatly assist in determining the true prevalence of heartworm and other pathogens present in the population. Heartworm prevention via repeated worming treatments is required from a young age to protect dogs against this parasite, however this is not feasable for a wild dingo population largely unaccesable to humans, and where feeding is also discouraged. Continued prohibition of domestic dogs on Fraser Island will assist in reducing the transfer of parasites and other pathogens (such as canine distemper or paramyxovirus and parvovirus) from mainland dogs to island dingoes.

We have shown that Fraser Island dingoes access and consume a wide variety of natural food items (Fig. 2) and potentially a reduced amount of anthropogenic food sources in comparison to previous studies (Table 1). We have also shown that they are in a generally healthy condition (Fig. 5). These findings improve our understanding of dingo ecology on the island, and have important conservation and management implications. Our results clearly reject hypotheses and anecdotal claims that dingoes have restricted diets and the general population is starving or is largely comprised of dingoes in poor physical condition^44^,^45^,^46^,^47^. The occurrence of anthropogenic food items appears to have decreased in dingo diets over the last 20 years (Table 1) in concert with targeted management actions to eliminate access to human food subsidies. Further work is needed to verify this, including a greater knowledge of the true sources of fish to dingoes, whether it is from humans or from naturally available sources. Future work should also include greater surveillance of parasites and pathogens that could potentially affect the viability of the island’s closed population of dingoes. Additional information on temporal fluctuations in food or prey availability, dingo diet and body weight may also alleviate some of the public concern expressed when a skinny dingo is observed amongst a typically healthy population. We conclude that there is nothing immediately alarming about the diet and physical health of dingoes on Fraser Island, but there remains an important need to continue monitoring these into the future. Such actions may assist in conserving the population of this important island top-predator.

## Methods

### Ethics statement

The dingo is considered native wildlife under the *Nature Conservation Act 1992*, and is protected in national parks, including Fraser Island (Great Sandy National Park). Elsewhere in Queensland dingoes are declared as a pest species under the *Land Protection (Pest and Stock Route Management) Act 2002*. Permission to enter the study site was granted by the Queensland Parks and Wildlife Service. All relevant procedures were approved under existing legislation and/or authorised by the Department of Environment and Resource Management (DERM) Animal Ethics Committee (permit numbers: DERM/2009/04/03, DERM2011/09/04, DERM2011/12/06 and SA2012/12/404), and the project was conducted in accordance with these approvals.

### Study site

Fraser Island (1,840 km^2^) is within the Great Sandy National Park located ~300 km north of Brisbane, off the south-east coast of Queensland, Australia (24°42’ S, 153 °15’ E). Fraser Island is a listed World Heritage site due to its unique natural values^85^. Dominant vegetation communities include sub-tropical rainforest and eucalypt woodlands, and the communities associated with freshwater lakes, wetlands and coastal beaches (Fig. 3). The sub-tropical climate varies in temperature from 22–29 °C in summer and 14–21 °C in winter (www.bom.gov.au). Mean annual rainfall varies across the island with ~1,200 mm falling coastally and ~1,800 mm falling inland. Approximately 500 residential dwellings are present on the island, and as a major tourism destination, Fraser Island attracts an additional ~400,000 visitors annually^86^,^87^.

### Dingo diet

Dingoes are present across the entire island^38^. Hence, the island was first divided into 26 sections of similar size (~70 km^2^) as a guide for collecting at least 30 scats from each section (to obtain a spatially consistent sample of scats from across the whole island). Scats were collected from roads, tracks, trails, around water sources and other focal points where dingoes were expected to defecate most frequently^4^,^60^,^88^. Searching occurred opportunistically across the period, but was predominately undertaken during several intensive surveys conducted between March 2011 and October 2014. Scats were confidently attributed to dingoes (and not other canids) because domestic dogs (*Canis familiaris*) are prohibited and not present on the island in National Park tenure except via occasional unauthorised visitation, and foxes (*Vulpes vulpes*) were first detected on the island (on a trial camera) in July 2012 and are presumably at extremely low densities. Scats were placed into paper envelopes, individually numbered and labelled with a description of the scat, its placement (e.g. road crossing, on a rise, on beach etc.), the date and GPS location. Scats were sent to a professional service provider who first sterilised and washed them before searching each scat for the remains of prey and other food items. Food items were identified to the lowest taxonomic level possible from hair, bone, scales, feathers and other diagnostic characters^65^,^66^. The occurrence of each food item was recorded for each scat and a percent volume of each food item within the scat was visually estimated using a grid system within the sorting tray.

Dingo stomach content samples, including material from the entire gastrointestinal tract, were collected opportunistically from dingoes euthanized or found deceased from other causes between August 2001 and January 2015^38^. Stomach contents were similarly analysed by Queensland Parks and Wildlife Service (QPWS) staff and veterinarians during necropsies, and food items identified were recorded as either absent or present. Both scat and stomach results are expressed as the ‘percent occurrence’ because many of the food items were detected infrequently^89^.

Additional records of food sources utilised by dingoes were obtained opportunistically from trail camera images, which occasionally included images of dingoes carrying some form of food or prey in their mouth. Images were also taken from cameras established to monitor dingo feeding behaviour at stranded and deceased whales (e.g. *Orcinus orca, Megaptera novaeangliae, Balaenoptera acutorostrata*), dolphins (e.g. *Tursiops* sp.), dugongs (*Dugong dugon*) and marine turtles (e.g. *Chelonia mydas, Caretta caretta, Eretmochelys imbricata*) washed ashore.

### Dingo body weight and condition

Dingo body weights were measured from deceased or euthanized animals, or those captured as part of routine dingo trapping and ear-tagging programs (additional details are found elsewhere^37^,^38^,^67^,^90^). When recaptures occurred very infrequently, the weight of the same dingo was sometimes measured again (i.e. the weights of a few individual dingoes were measured two or more times throughout their lives). Dingo ages (to the nearest year) were estimated by visual assessment of tooth eruption and wear, coat colouring, body size, breeding status, body condition and conformation or shape, noting the date of capture, death or euthanasia and the annual breeding cycle of dingoes^13^. All dingoes were further ascribed an assumed birth date of July 1^st^ (unless otherwise known from first-hand observation) for the year of birth assigned. Because annual whelping peaks in July^91^,^92^,^93^, this approach essentially estimates dingo ages to within a few weeks of their true age^38^.

External body condition of most captured, euthanized or deceased dingoes was assessed visually and by palpation of muscle bodies, and scored on a scale from 1 to 5 (1 = very poor condition or emaciated, 5 = grossly overweight or obese), as commonly used for domestic canids worldwide^81^,^82^. Internal body condition was assessed by examining the presence of fat in and around the heart, kidneys, mesentery, peri-renal areas and the pericardium, and also subcutaneous fat (along the centre of the abdomen) from a random sample of the deceased and euthanized dingoes. For heart and kidney fat, the percentage cover was first assessed, followed by the thickness of fat (mm) in covered areas. The thickness of subcutaneous fat was similarly measured. The presence of fat in the mesentery and peri-renal areas was categorised as either nil, minimum, minimal-moderate, moderate, abundant or excessive (Fig. 6).

### Ecto- and endoparasites

The prevalence of both ecto- and endoparasites was recorded for most dingoes either captured, euthanized or found deceased. Endoparasites were not sought from live dingoes, although a limited number of fresh scat samples (N = 44) were assessed for endoparasites by a veterinarian. The presence of endoparasites was typically assessed by a veterinarian during necropsy of euthanized or deceased dingoes, although obvious endoparasites (e.g. adult worm species and intestinal flukes) were assessed by QPWS staff.

Data recording practices varied over the period of data collection (since 2001), and hence weights, body scores, fat reserves and the presence of all parasites was not recorded on all possible occasions. Accordingly, data or information on each of these was not always available, and our sample sizes reflect the best available data. All maps were created new by the authors in ArcGIS v10.3 (ESRI Inc).

## Additional Information

**How to cite this article**: Behrendorff, L. *et al*. Insects for breakfast and whales for dinner: the diet and body condition of dingoes on Fraser Island (K’gari). *Sci. Rep*. **6**, 23469; doi: 10.1038/srep23469 (2016).

## Supplementary Material

Supplementary Information

## Figures and Tables

**Table 1:** Comparison of available studies reporting the diet of dingoes on Fraser Island (% occurrence).

	Moussalli1994†	Twyford1995^†^	Baker2004^†~^	Angel2006^†^	Currentstudy
*N scats*	*28*	*1073*	*86*	*126*	*2196*
Total mammal	39	53.8	–	79.4	85.1
Bandicoots	29	32.8	25.5	51.6	49.9
Rodents	11	39	8.9	32.5	10
Reptiles	0	1.6	8.5	11.1	14.9
Birds	25	4.2	2.4	5.6	7.4
Fish	61	25.6	53	19	26.8
Human sourced*	39	46.9^	10.3	26.2	7.5
Invertebrates	3	6.6	26	56.3	9.8
Vegetation	7	36.5	17	98.4	6

^Contains both human-sourced food and rubbish. *Not including fish frames. ^†^Unpublished studies. ~Values are unverified.

**Table 2:** Recorded marine dingo food items found stranded on Fraser Island beaches, January 2011 to December 2015 (see also Fig. S2).

Common name	Species name	N
Green turtle	*Chelonia mydas*	66
Hawksbill turtle	*Eretmochelys imbricata*	6
Loggerhead turtle	*Caretta caretta*	3
Olive ridley turtle	*Lepidochelys olivacea*	1
Unknown turtle	*Chelonia* sp.	4
Humpback whale	*Megaptera novaeangliae*	10
Orca	*Orcinus orca*	3
Melon-headed whale	*Peponocephala electra*	2
Minke whale	*Balaenoptera acutorostrata*	2
Sperm whale	*Physeter macrocephalus*	1
Pygmy sperm whale	*Kogia breviceps*	1
Cuvier’s beaked whale	*Ziphius cavirostris*	1
Pantropical spotted dolphin	*Stenella attenuata*	2
Indo-pacific bottlenose dolphin	*Tursiops aduncus*	2
Unknown dolphin	*Tursiops* sp.	5
Dugong	*Dugong dugon*	7
New Zealand fur seal	*Arctocephalus forsteri*	1
Total		117

**Table 3:** Prevalence of ecto- and endoparasites from dingoes on Fraser Island.

Source	Parasite	Frequency	N	%
External examination	Fleas	18	773	2.3
	Ticks	25	773	3.2
	Mange	1	773	<0.1
	Nil ectoparasites	730	773	94.4
Internal examination	Heartworm	10	149	6.7
	Total hookworm	34	111	30.6
	*Spirometra* spp.	9	111	8.1
	Total tapeworm	11	111	9.9
	Flukes	6	111	5.4
	Total nematodes	4	111	3.6
	Nil endoparasites	122	151	80.8
Fresh faeces	*Ancylostoma* spp.	7	44	15.9
	Total hookworm	39	44	88.6
	*Toxocara canis*	1	44	2.3
	Whipworm	1	44	2.3
	Total roundworm	2	44	4.5
	Nil endoparasites	4	44	9.1
