# Supplementary material for: Insects for breakfast and whales for dinner: the diet and body condition of dingoes on Fraser Island (K’gari)
Source: Sci Rep. 2016 Mar 24;6:23469. doi: 10.1038/srep23469 (PMC4806299; doi:10.1038/srep23469)
Supplement: Supplementary Information [file srep23469-s1.docx]

**Supplementary material**

The following supplementary material is associated with:

Behrendorff, L. *et al*. Insects for breakfast and whales for dinner: the diet and body condition of dingoes on Fraser Island (K’gari). *Sci. Rep.* **6**, 23469; doi: 10.1038/srep23469 (2016).

**Journal:** *Scientific Reports*

**Corresponding author:** Benjamin L. Allen, The University of Southern Queensland (email: [benjamin.allen@usq.edu.au](mailto:benjamin.allen@usq.edu.au))

**Contents:**

- Table S1
- Figure S1
- Figure S2

Table S1 – Food items found in dingo scats or stomachs, or observed in trail camera photos.

|  | **Food item** | **Taxonomic name** | **Scat** | **Stomach** | **Camera** |
| --- | --- | --- | --- | --- | --- |
| Terrestrial mammals | *Horse | *Equus caballus* | X |  |  |
|  | *Pig | *Sus scrofa* | X |  |  |
|  | Dingo (prey) | *Canus lupus dingo* | X |  |  |
|  | Dingo (grooming hair) | *Canus lupus dingo* | X |  |  |
|  | *Cat | *Felis catus* | X |  |  |
|  | Eastern grey kangaroo | *Macropus giganteus* | X |  |  |
|  | Swamp wallaby | *Wallabia bicolor* | X | X | X |
|  | Long-nosed potoroo | *Potorous tridactylus* | X |  | X |
|  | Mountain brushtail possum | *Trichosurus caninus* | X |  | X |
|  | Northern brown bandicoot | *Isoodon macrourus* | X | X | X |
|  | Long-nosed bandicoot | *Peremales nasuta* | X |  |  |
|  | Swamp rat | *Rattus lutreolus* | X |  |  |
|  | Unidentified rat | *Rattus* spp. | X | X |  |
|  | Water rat | *Hyrdromys chrysogaster* | X |  |  |
|  | *Black rat | *Rattus rattus* | X |  |  |
|  | Pale field rat | *Rattus tunneyi* | X |  |  |
|  | Bush rat | *Rattus fuscipes* | X | X |  |
|  | Eastern chestnut mouse | *Pseudomys gracillicaudatus* | X |  |  |
|  | *House mouse | *Mus musculus* | X |  |  |
|  | Delicate mouse | *Pseudomys delicatulus* | X |  |  |
|  | Unidentified rodent |  | X | X | X |
|  | Fawn-footed melomys | *Melomys cervinipes* | X |  |  |
|  | Grassland melomys | *Melomys burtoni* | X |  |  |
|  | Yellow-footed antechinus | *Antechinus flavipes* | X | X |  |
|  | Common dunnart | *Sminthopsis murina* | X |  |  |
|  | Short-beaked echidna | *Tachyglossus aculeatus* | X | X | X |
| Arboreal mammals | Sugar glider^ | *Petaurus breviceps* | X |  |  |
|  | Squirrel glider^ | *Petaurus norfolcensis* | X |  |  |
|  | Little red flying fox | *Pteropus scapulatus* | X |  |  |
|  | Black flying fox | *Pteropus alecto* | X |  |  |
|  | Unidentified flying fox |  | X |  |  |
|  | Grey-headed flying fox | *Pteropus poliocephalus* | X |  |  |
|  | Microbat |  | X |  |  |
|  | Unidentified hair/fur |  |  | X |  |
|  | Unidentified mammal |  |  |  | X |
|  | Unidentified bones |  | X | X |  |
|  | Unidentified meat/flesh/fat |  |  | X |  |
| Marine mammals | Sperm whale | *Physeter macrocephalus* |  |  | X |
|  | Humpback whale | *Megaptera novaeangliae* |  |  | X |
|  | Orca | *Orcinus orca* |  |  | X |
|  | Melon-headed whale | *Peponocephala electra* |  |  | X |
|  | Cuvier's beaked whale | *Ziphius cavirostris* |  |  | X |
|  | Indo-Pacific bottlenose dolpin | *Tursiops aduncus* |  |  | X |
|  | Pantropical spotted dolphin | *Stenella attenuata* |  |  | X |
|  | Unidentified dolphin |  |  |  | X |
|  | Dugong | *Dugong dugon* |  |  | X |
| Marine reptiles | Loggerhead turtle | *Caretta caretta* |  |  | X |
|  | Green turtle | *Chelonia mydas* |  |  | X |
|  | Hawksbill turtle | *Eretmochelys imbricata* |  |  | X |
|  | Olive ridley turtle | *Lepidochelys olivacea* |  |  | X |
|  | Unidentified turtle |  | X | X | X |
| Human-sourced food | HSF fish frames |  |  | X | X |
|  | HSF bread |  |  | X |  |
|  | HSF other |  |  | X |  |
|  | HSF grains |  |  | X |  |
|  | HSF meatbones |  |  | X |  |
|  | HSF pasta |  |  | X |  |
|  | HSF coconut |  |  | X |  |
|  | HSF vegetables |  |  | X |  |
|  | HSF fruit |  |  | X |  |
|  | HSF fishing bait |  |  | X |  |
| Other food items | Large skink |  | X |  |  |
|  | Goanna | *Varanus* spp. | X | X |  |
|  | Snake |  | X | X |  |
|  | Small skink |  | X | X |  |
|  | Agamid |  | X |  |  |
|  | Gecko |  | X |  |  |
|  | Bird |  | X | X |  |
|  | Fish |  | X | X |  |
|  | Crab |  |  | X |  |
|  | Ant/termite |  | X |  |  |
|  | Beetle |  | X | X |  |
|  | Unidentified invertebrate |  | X | X |  |
|  | Grasshopper |  | X |  |  |
|  | Centipede |  | X |  |  |
|  | Scorpion |  | X |  |  |
|  | Maggots |  |  | X | X |
|  | Crustacean |  | X | X |  |
|  | Mollusc |  | X | X |  |
|  | Seeds/fruit |  | X | X |  |
|  | Vegetation |  | X | X |  |
|  | Grass |  | X | X |  |
|  | Pumace |  |  | X |  |
|  | Sand |  |  | X |  |
|  | Rubbish |  | X | X | X |
|  | Other |  |  | X |  |


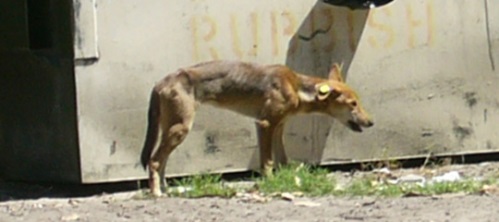

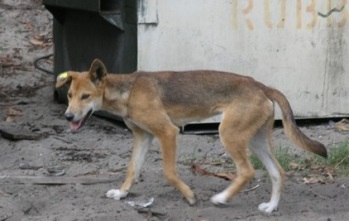

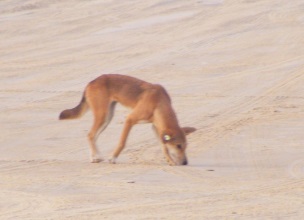

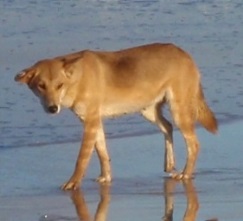


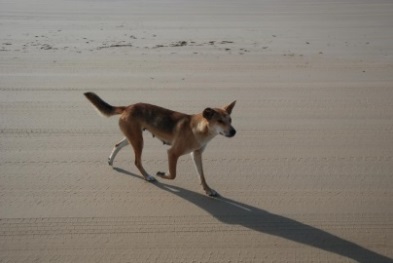

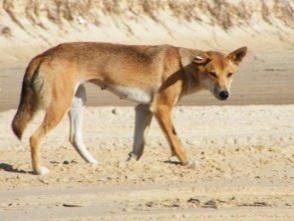

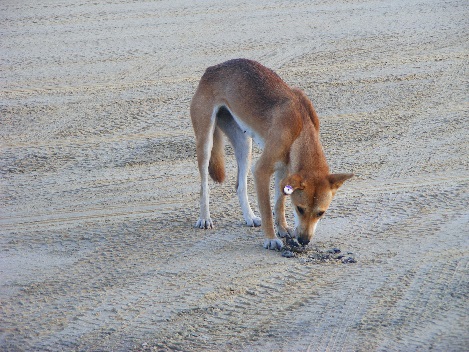

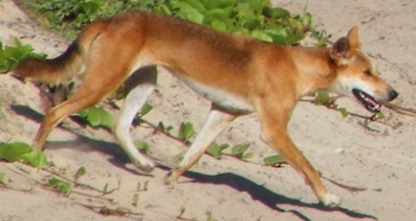


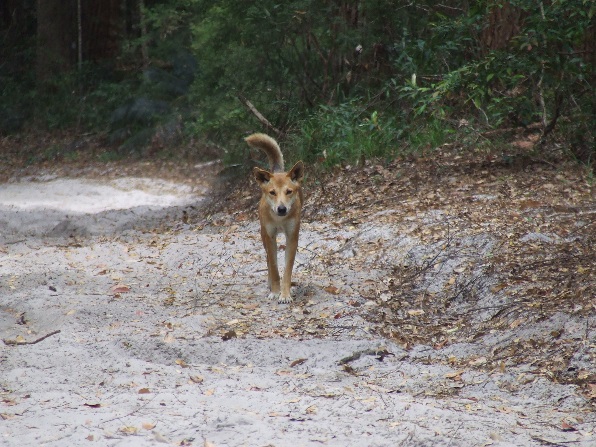

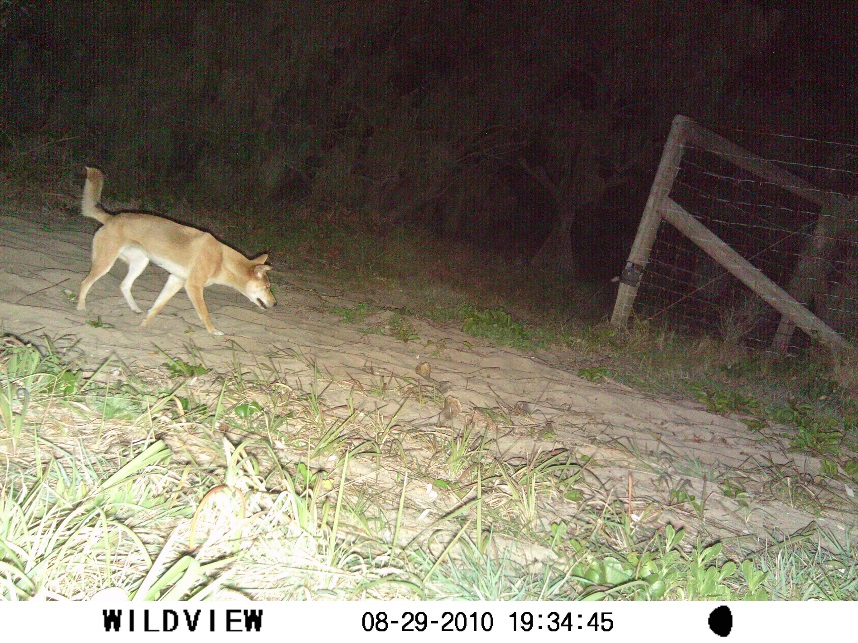

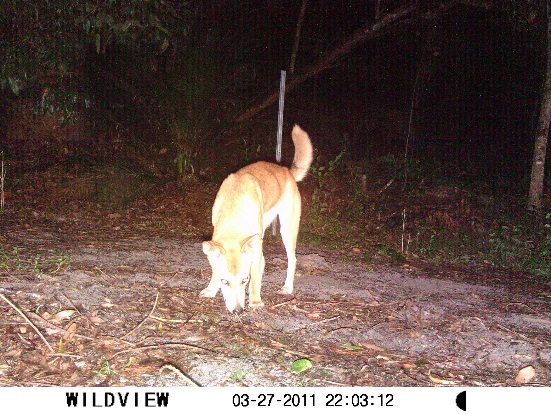

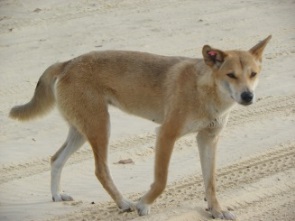

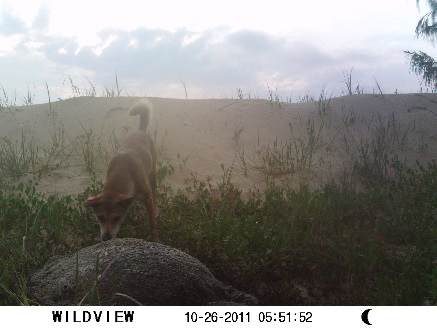


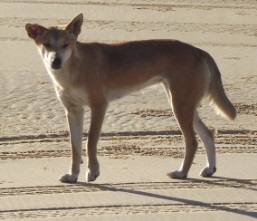

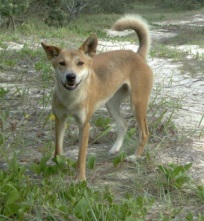

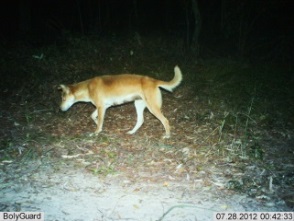

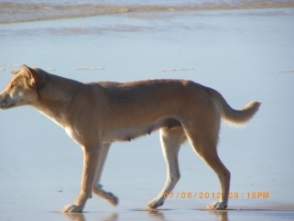

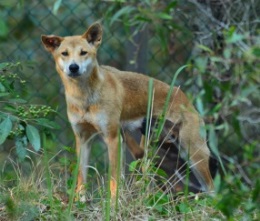


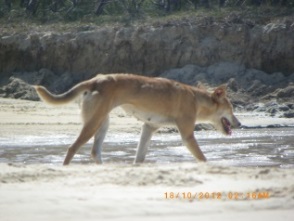

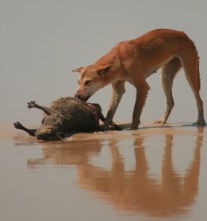

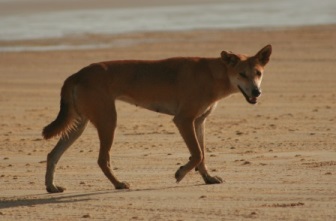


Fig. S1 – Photographs of the same individual female dingo taken between October 2008 and January 2013, showing temporal changes in coat colour and body condition (see also [Hesse 2010](#_ENREF_1)). This individual was born in July 2008 and was a commonly-observed resident dingo of the Eurong pack. She was ear-tagged a few months later (original Dingo ID: PuYY08f), was re-tagged in March 2011 (new Dingo ID: PiPiPi08f) after her original tag had been lost, and has not been observed (presumed deceased) since April 2013. In sequential order (from top left to bottom right) the photo dates are: October 2008, December 2008, March 2009, June 2009 (pregnant), August 2009 (lactating), September 2009, December 2009, January 2010 (tag missing), February 2010, August 2010* (pregnant), March 2011*, September 2011 (retagged), October 2011* (eating deceased turtle), November 2011, November 2011*, July 2012* (lactating), August 2012 (lactating), August 2012 (suckling pup), October 2012, December 2012 and January 2013.

* Denotes camera trap images.

Hesse, A., 2010. Fraser Island dingo population study: interim report Stage 1. Queensland Parks and Wildlife Service, Department of Environment and Resource Management, Eurong, Fraser Island.


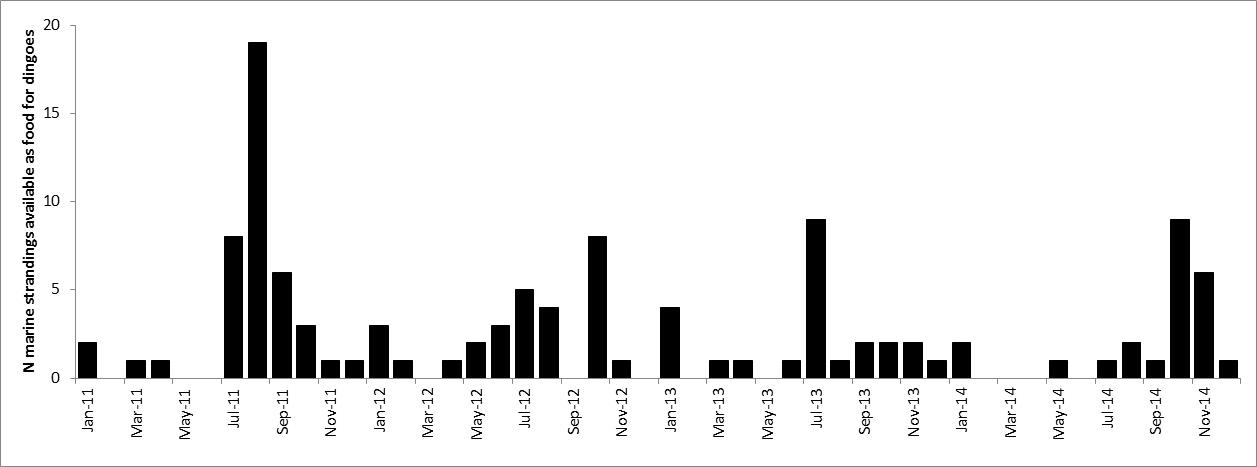


Fig. S2 – Trends in marine mammals and reptiles washed ashore between January 2011 and December 2014 (NB Does not include animals successfully released or rescued; see also Table 2).
